# Persistent delusional disorder: psychopathological remission associated with clozapine-induced epileptic seizures

**DOI:** 10.1192/j.eurpsy.2023.2286

**Published:** 2023-07-19

**Authors:** M. Felizardo, C. Freitas

**Affiliations:** Centro Hospitalar de Trás-os-Montes e Alto Douro, Vila Real, Portugal

## Abstract

**Introduction:**

Persistent delusional disorder has some features similar to schizophrenia, although the functionality of patients with this diagnosis is usually higher and is diagnosed at an older age. Although the pharmacological treatment of schizophrenia has been studied extensively, there is not much data on the treatment of persistent delusional disorder. Regarding the use of clozapines specifically in persistent delusional disorders, there are some case reports with encouraging results. Electroconvulsive therapy is not generally used as a treatment for persistent delusional disorder.

**Objectives:**

To reflect on the relevance of using electronvulsive therapy in the treatment of persistent delusional disorder.

**Methods:**

Through the description of a clinical case in which there was evidence of remission of resistant psychotic symptoms after clozapine-induced epileptic seizures, the authors hypothesize the existence of a direct relationship between the crisis and the resolution of a persistent delusional disorder.

**Results:**

A.F., 78 years old, male. No personal history of psychiatric or medical-surgical illness. Admitted for psychotic decompensation framed in persistent delusional disorder. The patient underwent pharmacological treatment with resistance to three lines of antipsychotics. With the introduction of clozapine 100mg/day, the patient had two epileptic seizures, followed by complete remission of psychotic symptoms.

**Conclusions:**

The clinical case described refers to a patient diagnosed with resistant persistent delusional disorder, with almost immediate resolution of the condition after epileptic seizures induced by clozapine. Taking into account the clinical response in our patient to two spontaneous epileptic seizures, we hypothesize that electroconvulsive therapy may be effective in the treatment of persistent delusional disorder.

**Disclosure of Interest:**

None Declared

